# Reconstruction of the Reaction of Andalusicin Lantibiotic Modification by Lanthionine Synthetase AncKC in a Heterologous Escherichia coli System

**DOI:** 10.32607/actanaturae.27347

**Published:** 2024

**Authors:** N. Z. Mirzoeva, S. O. Pipiya, Yu. A. Mokrushina, M. V. Serebryakova, A. A. Grigoreva, S. A. Dubiley, S. S. Terekhov, I. V. Smirnov

**Affiliations:** Shemyakin–Ovchinnikov Institute of Bioorganic Chemistry of the Russian Academy of Sciences, Moscow, 117997 Russian Federation; Department of Chemistry, Lomonosov Moscow State University, Moscow, 119991 Russian Federation; A.N. Belozersky Institute of Physicochemical Biology, Moscow, 119991 Russian Federation; Center of Life Sciences, Skolkovo Institute of Science and Technology, Moscow, 121205 Russian Federation; Institute of Gene Biology, Russian Academy of Sciences, Moscow, 119334 Russian Federation

**Keywords:** lanthipeptides, posttranslational modifications, antimicrobial peptides

## Abstract

The increasing resistance of microorganisms to antibiotics makes it a necessity that we search for new antimicrobial agents. Due to their genetically encoded nature, peptides are promising candidates for new antimicrobial drugs. Lantipeptide andalusicin exhibits significant antimicrobial activity against Gram-positive bacteria, making it a promising scaffold for the development of DNA-encoded libraries of lantibiotics. In this study, the modification reaction of andalusicin by class III lanthionine synthetase AncKC was reconstructed in a heterologous *Escherichia coli *system. The results obtained open possibilities for creating novel peptide- based antimicrobial agents.

## INTRODUCTION

Searching for novel antimicrobial agents is among the most pressing challenges faced by researchers in the 21^st^ century. The rapid spread of multidrug-resistant bacterial strains represents a huge risk to public health and needs particular attention. Peptides are attractive candidates for use as new antibiotics because of their genetically encoded nature, making it possible to generate a diverse group of potential artificial antimicrobial agents [1, 2].

Lantipeptides are a group of peptides that are produced by Gram-positive bacteria on ribosomes and undergo post-translational modifications. Posttranslational modifications of lantipeptides involve dehydration of Ser and Thr residues to dehydroalanine (Dha) and dehydrobutyrine (Dhb), followed by cyclization via the formation of intramolecular thioether bonds through the Cys residue, giving rise to the noncanonical amino acids lanthionine (Lan) and methyllanthionine (MeLan) [3]. Lantipeptides are synthesized as a prepeptide comprising the C-terminal sequence that undergoes post-translational modifications and the N-terminal leader peptide that is recognized by modifying enzymes and is then proteolytically removed [4]. Lantipeptides exhibiting antimicrobial activity, commonly referred to as lantibiotics, are of the greatest interest [5]. Classification of lantipeptides is based on the differences in the structures and mechanisms of action of the enzymes involved in the posttranslational modification of lantipeptides. Four classes of lantipeptides have been recognized until recently; however, the latest findings suggest that a new, fifth class, can exist [6].

**Fig. 1:**
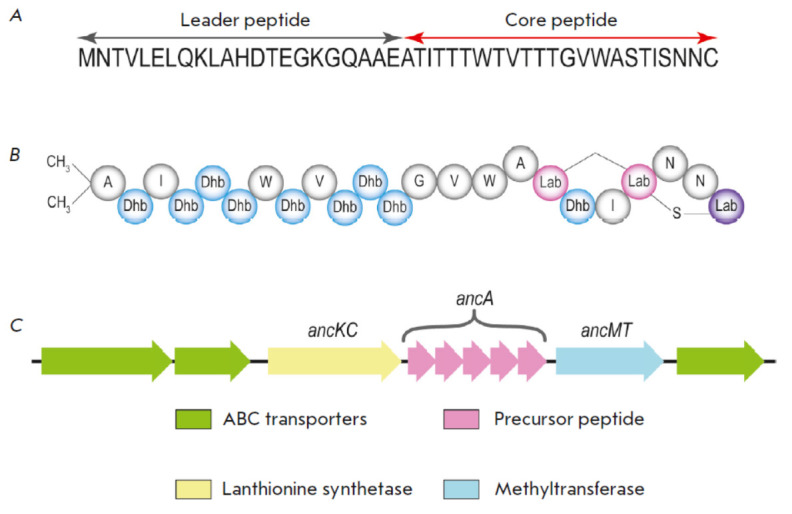
The structure of lantibiotic andalusicin: (*A*) the primary structure of andalusicin; (*B*) the structure of mature andalusicin; and (*C*) schematic representation of the andalusicin biosynthetic gene cluster

Lantipeptide andalusicin was isolated from the bacterium *Bacillus thuringiensis SV andalusiensis* B23193 (*Fig. 1*) [7]. The andalusicin biosynthesitic gene cluster contains genes encoding class III lanthionine synthetase AncKC involved in post-translational modification of lantipeptides, methyltransferase performing methylation, and ABC transporters export

## MATERIALS AND METHODS

**Plasmid construction and transformation of bacterial cells**

**Fig. 2:**
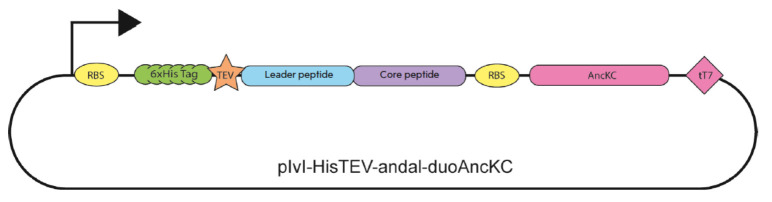
Schematic representation of the genetic construct containing the transcription unit for andalusicin biosynthesis

The *ancKC* and *ancA *genes were produced by chemical synthesis, treated with restriction endonucleases, and ligated into the pIvI-His-TEV vector (*Fig. 2*).

*E. coli *BL21 (DE3) cells were selected as a producer strain. The resulting genetic construct was used for chemical transformation of bacteria. Expression of the genes within the construct ensured co-expression of prepeptide and lanthionine synthetase AncKC, thus resulting in *in vivo *post-translational modifications. The transformed clones were transferred into Petri dishes with a 2xYt agar-based growth medium (16 g/L tryptone, 10 g/L yeast extract, and 5 g/L NaCl) supplemented with ampicillin to a concentration of 100 μg/mL.

**Production of modified andalusicin**

A solitary colony of the producer strain was selected for cultivation in 10 mL of the liquid 2xYT growth medium at 37°C overnight. The overnight bacterial culture (1 mL) was inoculated in 100 mL of liquid 2xYT growth medium. The cells were cultured at 37°C under shaking until *OD*600 = 0.4–0.6; IPTG was then added to a final concentration of 1 mM, and the cells were incubated at 18°C for 18 h under constant stirring. Upon expression completion, the cell mass was sedimented by centrifugation.

**Purification of recombinant modified andalusicin**

The cell mass was disintegrated ultrasonically and centrifuged at 12,000 rpm for 20 min. The following lysis buffer was used: 20 mM Tris-HCl pH 8.0, 500 mM NaCl. Andalusicin was purified by metal-chelate affinity chromatography using the Ni-NTA sorbent (Qiagen, Germany). An application buffer (20 mM Tris-HCl pH 8.0, 500 mM NaCl, 10 mM imidazole) and elution buffer (20 mM Tris-HCl pH 8.0, 500 mM NaCl, 300 mM imidazole) were used for chromatographic purification of the peptide. The samples after chromatographic purification were analyzed using denaturing Tricine–polyacrylamide gel electrophoresis under denaturing conditions [8].

**Mass spectrometry analysis**

To conduct mass spectrometry analysis, the samples were treated with iodoacetamide and subjected to trypsinolysis (Promega, USA). Next, 0.5 μL of the sample was mixed with 0.5 μL of a 2,5-dihydroxybenzoic acid solution (40 mg/mL in 30% acetonitrile, 0.5 TFA, Sigma Aldrich) on a target and dried.

The mass spectra were recorded on a MALDI-TOF mass spectrometer (Bruker Daltonics, Germany) in the positive ion mode. The spectra of proteolytic peptides were recorded using a reflectron; the accuracy of monoisotopic masses of singly charged protonated ions was ~ 0.005% (50 ppm). Tandem mass spectrometry fragmentation spectra were recorded with an accuracy in the measuring of fragmented ions ≥ 1 Da. Peptides were identified based on the combined peptide fingerprinting data and fragmentation spectra of individual peptides using the FlexAnalysis 3.3 and Biotools 3.3 software (Bruker Daltonics). The Mascot 2.3.02 software was used to search across the in-house database where the sequences of putative proteins had been preliminarily deposited, with allowance for the potential oxidation of Met residues with atmospheric air, potential modification of Cys residues with iodoacetamide or β-mercaptoethanol, potential dehydrogenation of Ser and Thr residues, and potential phosphorylation of Ser and Thr residues. Candidate proteins with score > 56 were considered to have been confidently identified (*p * < 0.05). The spectra were additionally marked manually.

**Analysis of antimicrobial activity**

Antimicrobial activity was analyzed using the agar diffusion technique in Petri dishes containing a 2xYT agar growth medium without the antibiotic. *B. cereus* ATCC 4342 colony was cultured in 10 mL of the liquid 2xYT growth medium at 37°C overnight. Bacterial lawn on the agar medium surface was produced using a cotton wool pad moistened with the overnight culture of the bacterium under study.

The sample of modified andalusicin precursor was treated with Glu-C protease (NEB, USA) to remove the leader sequence. The resulting reaction mixture was applied dropwise onto Petri dishes preliminarily divided into equal segments so that droplets contained 8, 4, 2, 1, and 0.5 μg of the core peptide of modified andalusicin. The Petri dishes were incubated at 37°C overnight, and zones of bacterial growth inhibition were detected.

## RESULTS AND DISCUSSION

Andalusicin, a class III lantipeptide, consists of 23 amino acid residues and carries the labionin ring, which is formed via post-translational modification of the precursor peptide by lanthionine synthetase AncKC. Andalusicin exhibits a strong antimicrobial activity against *B. cereus *ATCC 4342; thus, it is an interesting target for further research.

A genetic construct co-expressing the genes of andalusicin prepeptide and lanthionine synthetase AncKC in a single reading frame was obtained in this study. Chemical transformation of *E. coli *BL21 (DE3) cells resulted in the strain producing modified andalusicin prepeptide.

The modification profile of the produced andalusicin prepeptide was analyzed to prove successful reconstruction of the modification reaction. The purest product fractions were used to conduct a mass spectrometry analysis.

**Fig. 3:**
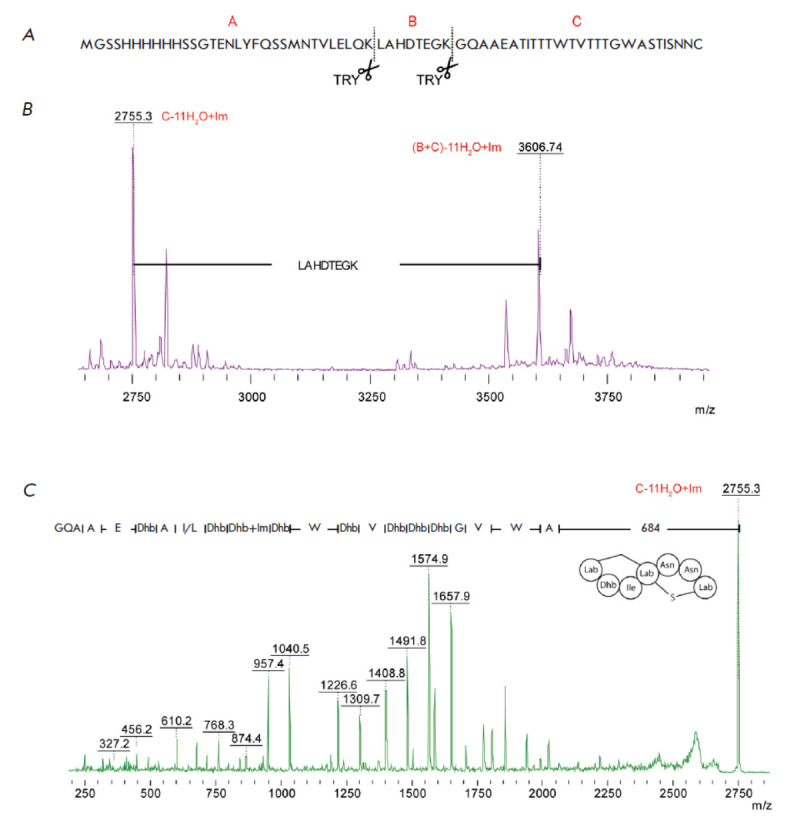
Mass spectrometric analysis of recombinant prepeptide andalusicin: (*A*) the structure of prepeptide andalusicin with indicated proteolysis sites; (*B*) the mass spectrum of prepeptide andalusicin after trypsin digestion; and (*C*) the fragmentation spectrum of the 2755.3 Da ion

The mass spectrometry analysis detected molecular ions with m/z 3,606.74 and 2,755.28 Da (*Fig. 3B*). The ions with m/z = 3,606.74 and 2,755.28 Da corresponded to the modified polypeptide trypsinolytically cleaved at the Lys31 (the B+C fragment) and Lys39 positions (fragment C), respectively. Fragmentation of fragment C ion (*Fig. 3C*) was used to further study the structure of andalusicin.

An analysis of the fragmentation spectrum of this molecular ion allowed us to determine the amino acid sequence of the modified peptide and identify 11 dehydroamino acid residues. The presence of Δm/z 683.84 Da in the fragmentation spectrum between the molecular ion and the first fragmented ion corresponds to the labionin ring at the C-terminus of andalusicin. The absence of fragmentation in this spectral region attests to the conformational rigidity of the structure.

**Fig. 4:**
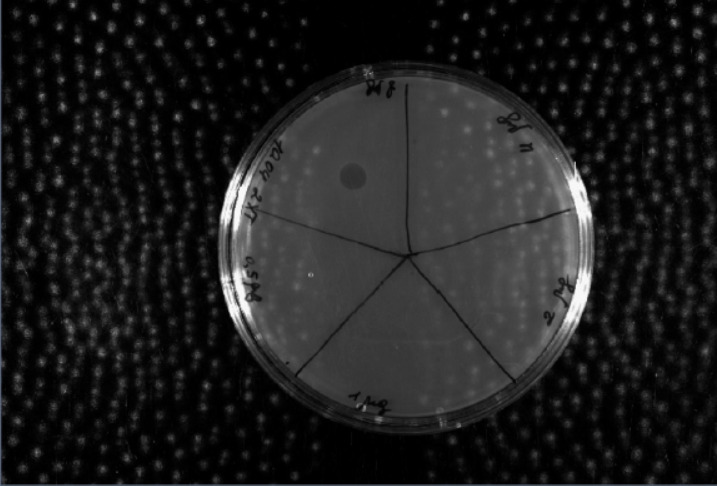
Results of the activity test of the obtained andalusicin analog using the agar diffusion method. The bacterial strain *B. cereus *ATCC 4342 was used as the model organism

The antimicrobial activity of an andalusicin analog was analyzed using the diffusion technique. The* B. cereus *ATCC 4342 strain was used as the model organism. The findings demonstrate that the antimicrobial activity is 20 ± 10-fold lower than that of mature wild-type andalusicin, which is possibly due to the fact that the andalusicin analog does not carry two methyl groups at the N-terminus of the core peptide (*Fig. 4*).

Hence, it has been demonstrated that AncKC introduces post-translational modifications into the andalusicin precursor corresponding to similar modifications to the wild-type peptide, which are caused by a successful reconstruction of this reaction in the heterologous* E. coli *system. The minimal antimicrobial activity is also indicative of successful modification of the andalusicin analog. The presence of dehydroamino acids within the structure, in combination with the labionin ring at the C-terminus of the lantipeptide, ensures binding to lipid II, one of the key participants in the bacterial cell wall biosynthesis.

The results of this study deepen our understanding of class III lantipeptides and open up new avenues for the targeted modification of recombinant antimicrobial peptides.

## CONCLUSIONS

Designing novel antimicrobial agents is among the top priorities of our time. Peptides are promising candidates for use as new antibiotics. In particular, some members of the lantipeptide family exhibit a strong antimicrobial activity; along with unique post-translational modifications, it makes them interesting study objects. We have generated a genetic construct co-expressing the genes of class III lanthionine synthetase AncKC and lantipeptide andalusicin, which enables simultaneous production and modification of the peptide in the heterologous protein producer *E. coli *BL21 (DE3). A mass spectrometry analysis of the recombinant andalusicin prepeptide verified that post-translational modifications corresponding to those in wildtype andalusicin were introduced. These findings broaden the scope of opportunity in using lantipeptides to search for and design novel antibiotics.
